# GLP-1 and the cardiovascular system

**DOI:** 10.1172/JCI194748

**Published:** 2026-02-16

**Authors:** Florian Kahles, Andreas L. Birkenfeld, Nikolaus Marx

**Affiliations:** 1 Department of Internal Medicine I, University Hospital Aachen, RWTH Aachen, Aachen, Germany.; 2 German Center for Diabetes Research (DZD), Neuherberg, Germany.; 3 Department of Internal Medicine IV, Diabetology, Endocrinology and Nephrology, Eberhard-Karls University Tübingen, Tübingen, Germany.; 4 Institute for Diabetes Research and Metabolic Diseases of the Helmholtz Center Munich, Tübingen, Germany.; 5 School of Cardiovascular and Metabolic Medicine and Sciences, Faculty of Life Sciences and Medicine, Kings College London, London, United Kingdom.

## Abstract

The incretin hormone glucagon-like peptide-1 (GLP-1) exerts potent effects on glucose metabolism, prompting the development of therapeutic strategies that enhance activity of the GLP-1 receptor (GLP-1R) pathway. Inhibitors of dipeptidyl peptidase 4 (DPP-4) prolong the half-life of endogenous GLP-1 and typically achieve reductions in HbA1c of 0.5%–0.8%. However, large-scale cardiovascular (CV) outcomes trials (CVOTs) with DPP-4 inhibitors demonstrated CV safety but did not show a reduction in CV events. A second incretin-based therapeutic approach was the development of GLP-1R agonists (GLP-1RAs). Various GLP-1RAs, including liraglutide, semaglutide, and dulaglutide, demonstrated a reduction in CV outcomes in large CVOTs. Initially, these medications were only available as injectable agents for subcutaneous administration, but recent technological advancements have enabled the development of orally available GLP-1RAs. A third incretin-based approach is tirzepatide, a dual agonist of GLP-1R and glucose-dependent insulinotropic polypeptide receptor (GIPR), which achieves greater HbA1c reduction and weight loss compared with GLP-1RAs alone. Ongoing large-scale CVOTs will determine its effects on hard cardiovascular endpoints. This Review summarizes the effects of GLP-1 and GLP-1RAs in the CV system as well as clinical data of GLP-1RAs in individuals with CV disease or high CV risk.

## Cardiovascular effects in experimental models

### Effects on atherosclerosis.

**Figure 1:**
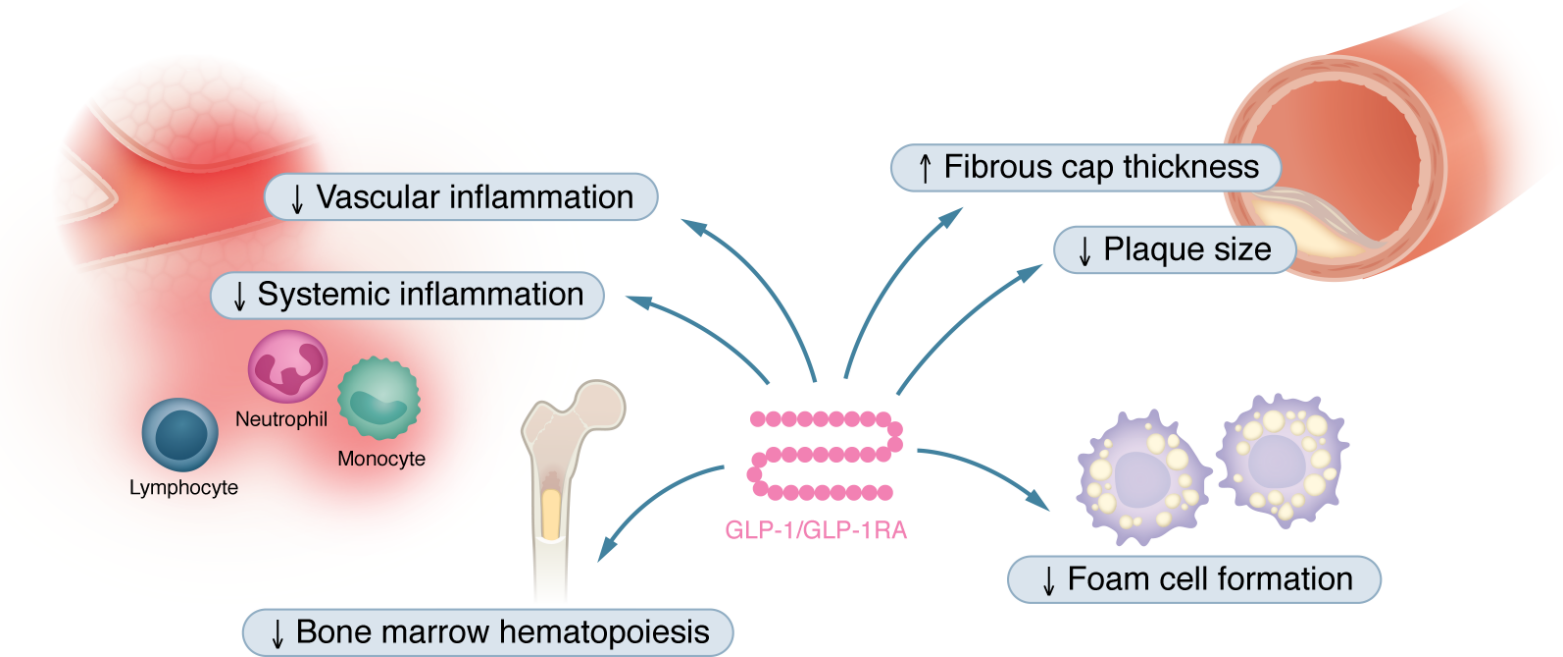
The effects of GLP-1 and GLP-1RAs on atherosclerosis. Abundant evidence indicates that GLP-1RAs protect against atherosclerosis independently of their effects on cholesterol, blood glucose, and body weight. In animal models, GLP-1RAs decrease both systemic and vascular inflammation, decrease hematopoietic stem cell proliferation, and reduce foam cell formation. Collectively, these effects result in reduced plaque size and increased fibrous cap thickness, indicating plaque stability.

Direct vascular effects of both native glucagon-like peptide-1 (GLP-1) and GLP-1 receptor agonists (GLP-1RAs) have been extensively studied in preclinical mouse models of atherosclerosis (Figure 1). For example, Nagashima and colleagues showed that administration of active GLP-1(7–36) amide via osmotic minipumps for 4 weeks decreased atherosclerotic lesions and macrophage numbers in the aortic wall in *Apoe^–/–^* mice on high-fat diet (HFD), compared with vehicle controls (1). The effects were inhibited by coinfusion with exendin(9–39), a specific antagonist of the GLP-1R, indicating that active GLP-1 exerts its vascular protective effects through the classical GLP-1R, which is expressed in vascular smooth muscle cells and endothelial cells. The antiatherosclerotic effects of GLP-1(7–36) amide were associated with a reduction in foam cell formation and downregulation of CD36 and acyl-coenzyme A:cholesterol acyltransferase-1 (ACAT-1) in macrophages. Importantly, GLP-1(7–36) treatment did not affect body weight, systolic blood pressure, or plasma concentrations of total cholesterol, HDL-cholesterol, and triacylglycerol (1). In another study, overexpression of active GLP-1(7–37) for 12 weeks in *Apoe^–/–^* mice on HFD using a hepatocyte-targeted adeno-associated viral vector system (2) reduced plaque macrophage content and plaque MMP-9 expression, and increased plaque collagen content and fibrous cap thickness without affecting overall lesion size. Importantly, overexpression of the GLP-1 metabolites GLP-1(9–37) and GLP-1(28–37), which do not activate the classical GLP-1R, showed similar vascular protective effects, suggesting receptor-independent effects of GLP-1 (3). In summary, these findings indicate direct vascular protective effects of active GLP-1 and its metabolites.

Similar vascular protective effects have been found in atherosclerosis-prone mice after treatment with GLP-1RAs. Treatment of *Apoe^–/–^ Irs^+/–^* mice (a mouse model of insulin resistance, metabolic syndrome, and atherosclerosis) with either lixisenatide or liraglutide decreased atherosclerotic lesion size, generated more stable plaques with fewer lesional leukocytes, and resulted in reduced necrotic cores and thicker fibrous caps (4). Consistent with this effect, dulaglutide reduced atherosclerotic lesion size in *Apoe^–/–^* mice with streptozotocin-induced diabetes (5). The protective effects of GLP-1RAs on atherosclerosis have also been confirmed in mice without diabetes. Continuous infusion of exendin-4 over 28 days reduced monocyte adhesion to the endothelium, downregulated aortic mRNA expression of intercellular adhesion molecule-1 (ICAM-1), and suppressed atherogenesis in *Apoe^–/–^* mice on HFD without affecting LDL-cholesterol (LDL-C) levels (6). Exendin-4 treatment for 8–12 weeks also reduced atherosclerosis (7, 8) and plaque macrophages (8) in *Ldlr^–/–^* mice on a high-cholesterol diet (HCD) (6). Likewise, multiple studies have reported a reduction in plaque size in atherosclerosis-prone mice treated with liraglutide (9–14). The vascular protective effects of liraglutide were associated with improved plaque stability (14), attenuated endothelial cell dysfunction (14, 15), reduced ICAM-1 expression in the aortic endothelium (12, 15), decreased leukocyte rolling on the endothelium and infiltration of myeloid cells into the vascular wall (13, 16), suppressed foam cell formation (13), and inhibition of vascular smooth muscle cell proliferation (10). In *Ldlr^–/–^* mice with uremia, which is known to increase the risk of atherosclerosis and cardiovascular (CV) death (17), Bisgaard et al. showed that liraglutide treatment for 11 weeks markedly reduced plaque area (18).

In addition to liraglutide, various studies have investigated the effects of semaglutide on atherosclerosis formation and vascular inflammation. Subcutaneous administration of semaglutide for 12–14 weeks in *Apoe*^–/–^ mice and 17 weeks in *Ldlr*^–/–^ mice decreased atherosclerotic lesion size and carotid intima-media thickness, a marker of subclinical atherosclerosis, while cholesterol levels were unaffected (9). Semaglutide partially prevented Western diet–induced gene expression changes in atherosclerosis pathways, including leukocyte recruitment; chemokine (C-C motif) ligand 2 (CCL2); leukocyte rolling, adhesion, and extravasation; cholesterol metabolism and lipid-mediated signaling; prostaglandin I_2_ synthase; extracellular matrix protein turnover; and plaque hemorrhage (9). These findings were confirmed in high-fat, high-cholesterol (HFHC) diet–fed mice with functional LDL receptor deficiency induced by viral expression of proprotein convertase subtilisin/kexin type 9–adeno-associated virus (PCSK9-AAV), in which daily administration of semaglutide for 18 weeks reduced aortic plaque area (11).

Taken together, these studies indicate that GLP-1 and GLP-1RAs protect against atherosclerosis by reducing vascular inflammation independently of cholesterol, blood glucose, and body weight. However, the exact underlying mechanisms are not fully understood. Interestingly, growing evidence suggests that GLP-1RAs reduce systemic inflammation beyond local antiinflammatory effects in atherosclerotic plaques. Exendin-4–treated *Ldlr*^–/–^ mice on an HCD not only showed smaller atherosclerotic lesions but also a more than 50% reduction in blood leukocytes, including Ly-6C^hi^ monocytes and neutrophils (7), suggesting downregulation of bone marrow hematopoiesis in response to activation of the GLP-1R. Indeed, exenatide treatment decreased bone marrow hematopoietic stem and progenitor cell proliferation in *Ldlr*^–/–^ mice on HFD (8) and in wild-type mice exposed to chronic-restrain stress (19). In another study, administration of semaglutide prior to LPS decreased levels of the proinflammatory cytokines TNF-α and IFN-γ and reduced circulating levels of the chemoattractant osteopontin (OPN) (9). These data suggest that GLP-1R agonism attenuates atherosclerosis formation and vascular immune cell activation most likely by reducing systemic inflammation independently of changes in lipid and glucose metabolism. Future studies are required to address remaining open questions, including the identity of the target cells on which GLP-1 acts to downregulate systemic inflammation and atherosclerosis formation. Elegant studies by McLean et al. showed that reduction of atherosclerosis and systemic inflammation in response to semaglutide was preserved in mice lacking the GLP-1R in endothelial and hematopoietic lineage cells (11). Based on Immgen (https://www.immgen.org/) and previous studies (7, 11, 20), most immune cells except for gut intraepithelial T lymphocytes show only very low expression of the classical GLP-1R. Furthermore, activation of the GLP-1R inhibits vascular smooth muscle cell proliferation (10); however, the extent to which smooth muscle cells contribute to the antiatherosclerotic properties of GLP-1 remains to be determined. Conditional knockout mouse models targeting the GLP-1R in smooth muscle cells will be needed to address this issue. Taken together, these studies suggest that further research is required to clarify the relevance of endothelial cells, smooth muscle cells, and immune cells, and whether the vascular protective and antiinflammatory effects of GLP-1 and GLP-1RAs are at least in part mediated through GLP-1R–independent effects.

### Effects on heart failure.

**Figure 2:**
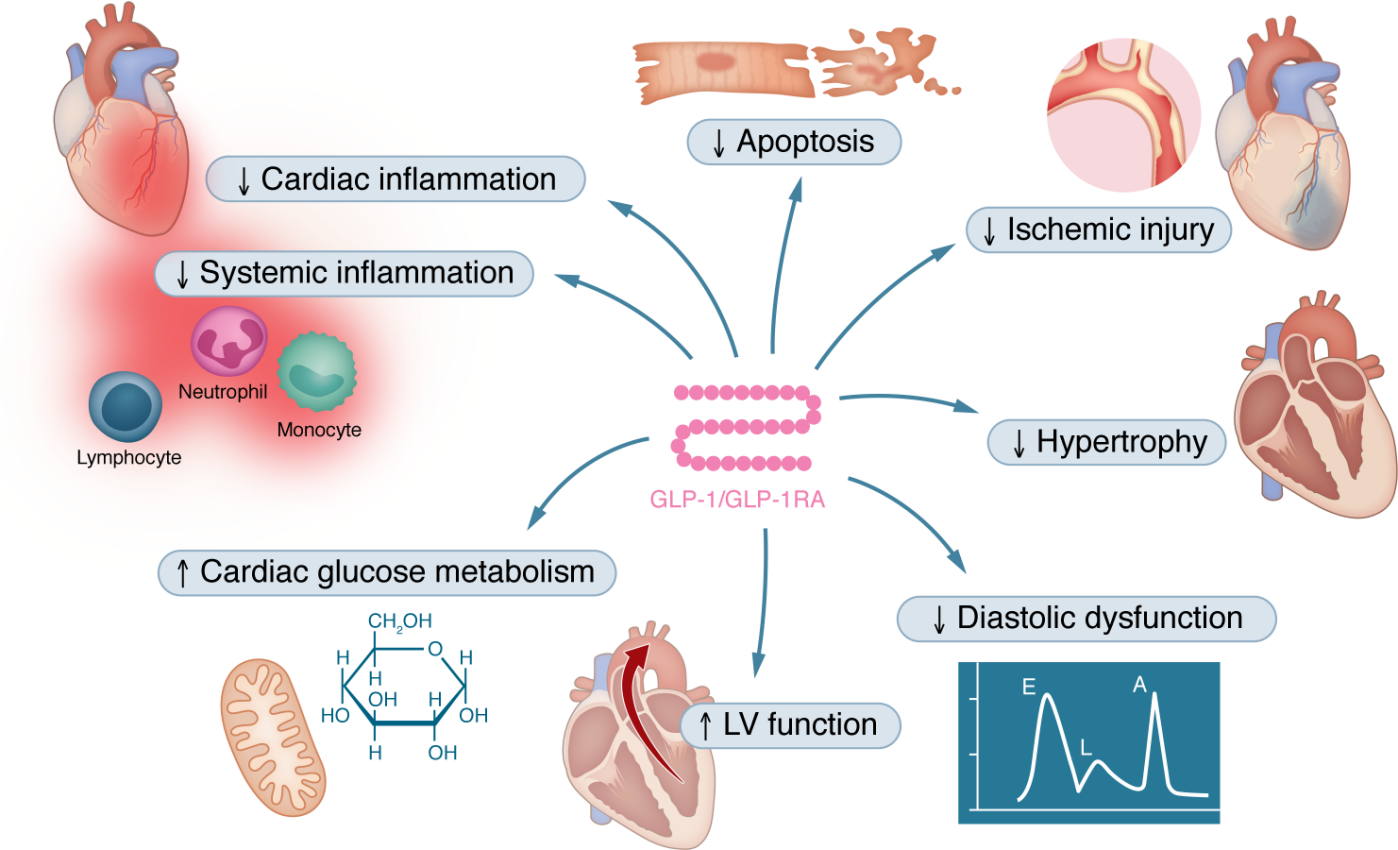
The effects of GLP-1 and GLP-1RAs on heart failure. In animal models of heart failure, GLP-1RAs prevent apoptosis of cardiomyocytes, reducing ischemic injury and preserving left ventricle function. Furthermore, GLP-1RAs protect against overload-induced hypertrophy and improve diastolic function in mouse models. GLP-1RAs promote glucose utilization in the myocardium and decrease systemic and cardiac inflammation.

In addition to vascular protective effects, GLP-1R agonism improves experimental heart failure across several species, including mice (21–32), rats (33–37), pigs (38, 39), and dogs (40, 41) (Figure 2). In lean Ossaba swine, systemic infusion of GLP-1(7–36) showed no cardiometabolic or hemodynamic effects prior to ischemia, but it increased cardiac output by approximately 2 L/min relative to vehicle controls during ischemia (39). In another study with obese Ossabaw swine, liraglutide treatment improved cardiac output in response to sympathomimetic challenge (dobutamine 0.3–10 μg/kg/min) 30 days after occlusion of the left anterior descending (LAD) artery (38). Since hearts from liraglutide-treated swine showed decreased β1-adrenoreceptor expression, these data suggest that activation of the GLP-1R might enhance cardiac output by inhibition of adverse sympathetic signaling in obese swine after myocardial infarction (MI) (38). In mice, pretreatment with liraglutide prior to permanent LAD artery ligation led to improved survival and reduced infarct size and augmented cardiac output. These effects were similar in mice with or without diabetes and were independent of weight loss. On a mechanistic level, liraglutide influenced both the expression and functional activity of cardioprotective genes in the murine heart, including Akt, GSK3β, PPARβ/δ, Nrf2, and HO-1 (21). Moreover, liraglutide (33), dulaglutide (34), and semaglutide (23) have been shown to attenuate myocardial ischemia/reperfusion injury, associated with inhibition of necroptosis by activating the PI3K/Akt pathway (33) and with inhibiting ferroptosis via activation of the PKC/S100A9 axis (23). Further studies found that liraglutide treatment inhibits cardiac and systemic inflammatory responses 3 hours after MI in mice, and that its cardioprotective effects require the GLP-1R in endothelial and hematopoietic (Tie2^+^) cell populations (22), but not in cardiomyocytes (42). These findings suggest that endogenous cardiomyocyte GLP-1R activity is not required for GLP-1RA–induced cardioprotection.

Another study investigated the endogenous GLP-1 system during acute MI in mice. Diebold et al. showed that an acute MI increases GLP-1 secretion within 6 hours. This MI-induced GLP-1 secretion was cardioprotective, as mice treated with a GLP-1R inhibitor had worse left ventricular (LV) function after LAD artery ligation. These findings suggest a counter-regulatory control system in which MI-induced inflammatory GLP-1 secretion inhibits cardiac dysfunction by increasing AMPK activity and stimulation of the mitochondrial respiratory capacity of noninfarcted tissue areas (25). Overall, these findings showed that GLP-1RAs across multiple species improve cardiac output during ischemic stress, reduce infarct size, dampen sympathetic and inflammatory signaling, and activate cardioprotective pathways such as PI3K/Akt and PKC/S100A9, thereby improving post-MI cardiac function and survival.

Similar to the antiinflammatory effects, it remains incompletely understood whether these cardioprotective effects of GLP-1 and GLP-1RAs are mediated at least in part independently of the classical GLP-1R. Interestingly, GLP-1(28–36) amide, a metabolite that does not bind to GLP-1R, was found to protect against cardiac injury in mice after LAD artery ligation by a mechanism involving mitochondrial trifunctional protein-α (MTPα) and intracellular soluble adenylyl cyclase (sAC) in cardiac smooth muscle and endothelial cells, but not cardiomyocytes (24). Future studies will be needed to elucidate the functional and clinical relevance of GLP-1R–independent effects of GLP-1 analogs.

In addition to ischemic cardiomyopathy, GLP-1 has also been shown to be protective in other types of heart failure. In conscious dogs with pacing-induced dilated cardiomyopathy, 48-hour infusion of recombinant GLP-1 improved LV performance, mediated by increased myocardial glucose uptake via p38α MAP kinase–mediated, nitric oxide–dependent mechanisms (40, 41). In addition, chronic GLP-1 administration prolonged survival in obese, hypertensive rats susceptible to spontaneous hypertensive heart failure. Mechanistically, GLP-1 preserved LV function and LV mass, promoted myocardial glucose utilization, and attenuated cardiomyocyte apoptosis (35). Liraglutide was found to be protective against angiotensin II– and pressure overload–induced cardiac hypertrophy via PI3K/Akt1 and AMPKα signaling in mice (43). Protective effects during pressure overload–induced cardiac hypertrophy have also been reported for semaglutide in rats that underwent transverse aortic constriction. In that study, semaglutide treatment improved cardiac mitophagy to suppress the activation of the NLRP3 inflammasome (36).

In rodent models of obesity and diabetes, liraglutide reduced myocardial TNF-α expression and NF-κB translocation by activating AMPK signaling (29) and augmenting myocardial glucose oxidation (30). In two studies, Withaar et al. examined the effects of liraglutide and semaglutide in a multifactorial mouse model of heart failure with preserved ejection fraction (HFpEF) induced by HFD and angiotensin II administration (31, 32). Mechanistically, liraglutide and semaglutide decreased LV hypertrophy and fibrosis, and improved diastolic dysfunction, lung congestion, and exercise capacity. Indeed, the cardioprotective effects of GLP-1RAs exceeded those of dietary weight loss, indicating direct and weight-loss-independent cardiac effects of liraglutide and semaglutide in HFpEF (31, 32). Future studies are needed to improve our understanding of the underlying molecular pathways. A recent study by Kuo et al. showed that liraglutide downregulates expression of the fibrosis markers TGF-β1 and collagen types I and III, which might explain, at least in part, how GLP-1RAs reduce cardiac fibrosis in heart failure models (44).

In summary, current evidence clearly shows direct cardioprotective effects of GLP-1 and GLP-1RAs in preclinical models of heart failure (Figure 2). However, our understanding of GLP-1R expression and downstream signaling in the myocardium across species is incomplete. In humans, GLP-1R expression has been found in atrial and ventricular tissue (45, 46), including cardiomyocytes (22). In contrast, in murine models *Glp1r* mRNA transcripts are predominantly found in isolated atrial cardiomyocytes, with no expression detected in ventricular cardiomyocytes (47). Future studies will be required to delineate the cellular localization of GLP-1R protein or GLP-1 binding sites within the heart and to define the underlying molecular pathways.

## Effects of GLP-1RAs in clinical trials

### Clinical studies with focus on atherosclerotic CV disease.

GLP-1RAs have transformed type 2 diabetes (T2D) treatment, offering not only robust glycemic control but also CV protection (48). Atherosclerotic CV disease (ASCVD) — including MI, stroke, and CV death — remains the leading cause of morbidity and mortality in patients with T2D (49). Multiple CV outcomes trials (CVOTs) have provided strong evidence that GLP-1RAs reduce ASCVD events (50–57), although the mechanisms remain multifaceted and are still under investigation.

Eleven large CVOTs have investigated the impact of GLP-1RAs on major adverse cardiovascular events (MACE) — defined as a composite of CV death, nonfatal MI, and nonfatal stroke — or on maximum walking distance in peripheral artery disease (57, 58). These trials include ELIXA, LEADER, SUSTAIN-6, EXSCEL, HARMONY, REWIND, PIONEER-6, AMPLITUDE-O, SELECT, SOUL, and STRIDE (50–54, 56, 57, 59, 60). Most of these trials used subcutaneous injections, although PIONEER-6 and SOUL evaluated an oral formulation of semaglutide (55, 60) (Table 1).

A meta-analysis of these major CVOTs demonstrated a 14% relative risk reduction in 3-point MACE (HR, 0.86; 95% CI, 0.80–0.93; *P* < 0.001), corresponding to a number needed to treat of 65 over several years to prevent one event, with moderate statistical heterogeneity (61). Upon exclusion of the ELIXA trial — which utilized the short-acting GLP-1RA lixisenatide and was associated with neutral CV outcomes — the relative risk reduction improved to 15% (HR, 0.85; 95% CI, 0.80–0.90; *P* < 0.001), and heterogeneity across the trials was substantially diminished (61). These findings underscore a robust and consistent CV benefit across longer-acting GLP-1RAs.

The recently completed SOUL trial further elucidated the CV benefits of oral semaglutide, addressing the limitations observed in the earlier PIONEER-6 trial, where a reduction in MACE did not reach statistical significance (55). In contrast, SOUL was sufficiently powered to rigorously evaluate superiority, demonstrating a significant 14% relative risk reduction in MACE (HR, 0.86; 95% CI, 0.77–0.96; *P* = 0.006). The number of events was 668 in the placebo group compared with 579 in the oral semaglutide group, supporting a consistent CV benefit (60). Importantly, these findings remained robust even after analyzing and adjusting for concomitant use of SGLT2 inhibitors, underscoring the independent contribution of oral semaglutide to CV risk reduction (62).

The SELECT trial evaluated whether once-weekly subcutaneous semaglutide could reduce MACE in individuals with overweight or obesity and established ASCVD but without T2D. In this large-scale trial of over 17,500 participants, the mean duration of exposure to semaglutide or placebo was 34.2 ± 13.7 months and the mean follow-up was 39.8 ± 9.4 months. The primary endpoint occurred in 569 of 8803 participants (6.5%) in the semaglutide group compared with 701 of 8801 participants (8.0%) in the placebo group, corresponding to an HR of 0.80 (95% CI, 0.72–0.90; *P* < 0.001) (59). These results provide compelling evidence that semaglutide reduces ASCVD risk also in patients with obesity and established ASCVD but not T2D, laying groundwork for novel therapeutic strategies for the treatment of obesity in people with high risk for CV events.

The CV benefits of GLP-1RAs cannot be fully attributed to glycemic control alone (63). As noted above, several lines of evidence suggest that these agents exert pleiotropic effects on vascular biology, inflammation, lipid metabolism, and more (48, 64, 65). An important observation from CVOTs in patients with T2D is that event curves for MACE diverge after 12–18 months of treatment, indicating that sustained exposure is required to realize benefits in ASCVD (48). The reductions in MI, stroke, and CV death suggest a targeted impact on atherosclerotic processes, rather than acute hemodynamic effects. However, GLP-1RAs also modestly reduce systolic blood pressure (SBP) by 2–4 mmHg (66). Although blood pressure lowering is an established mechanism for reducing MACE, the magnitude seen with GLP-1RAs is likely insufficient to explain the full extent of CV benefit (66). For example, SBP reductions were only 1.2 mmHg in the LEADER study and 1.7 mmHg in the REWIND study (54, 67), yet, the GLP-1RA–mediated reduction in ASCVD reduction was comparable to that observed in the other CVOTs (48). Similarly, GLP-1RAs induce small reductions in total cholesterol, LDL-C, and triglycerides, but these are also unlikely to fully account for ASCVD benefits (68). GLP-1RAs reduce HbA1c by 0.8%–1.5% points in people living with T2D and 0.3% points in people with obesity without T2D, depending on baseline levels (48, 49, 59, 69–71). However, previous large-scale trials comparing intensive versus standard glycemic control, such as ACCORD and ADVANCE, demonstrated that while intensive glucose lowering modestly reduced the incidence of nonfatal MI, it did not significantly reduce CV mortality among patients with T2D (70, 72, 73). Of note, those trials did not use GLP-1RAs to improve glycemic control. It remains uncertain whether the lack of improvements in CV death associated with intensive glycemic control in individuals with T2D also extends to those with prediabetes or to interventions involving newer pharmacological agents such as GLP-1RAs and SGLT2 inhibitors (74–77). Several lines of evidence suggest that bringing prediabetic (intermediate hyperglycemia) glucose values back to normoglycemia may have beneficial effects (74, 78, 79), a notion that clearly needs to be formally proven.

Weight loss achieved with GLP-1RAs ranges from 2% to 5% in most CVOTs, including CVOTs with injectable semaglutide (52, 60) using 1.0 mg/week as the target dose. However, in clinical practice higher doses up to 2.0 mg are used in patients with T2D, and up to 2.4 mg/week in patients with obesity without T2D. Notably, in the SELECT trial, individuals with obesity with established ASCVD without T2D were treated with semaglutide 2.4 mg/week and demonstrated a weight loss of over 9% (59). In this trial, the relative MACE risk reduction was 20% (HR, 0.80; 95% CI, 0.72–0.90; *P* < 0.001). In contrast, the mean relative risk reduction in studies involving individuals with T2D, where weight loss was between 2% and 5%, was 15%, as seen in the aforementioned trials (48, 57, 59, 71). Additionally, the GLP-1R/glucose-dependent insulinotropic polypeptide receptor (GIPR) dual agonist tirzepatide can induce weight loss of 10%–20%, and the results of the ongoing SURPASS-CVOT trial may provide more clarity on whether weight loss itself is an independent driver of CV protection (74). Moreover, it is increasingly recognized that weight loss itself primarily serves as a surrogate marker for favorable changes in body fat distribution, particularly reductions in ectopic fat depots such as visceral adipose tissue and intrahepatic lipid content, both of which are strongly associated with ASCVD risk (80, 81). In support of this, 2.4 mg/week of semaglutide in the SELECT trial led to a substantial reduction in waist circumference by nearly 8 cm, reflecting decreased visceral adiposity (59). Similarly, high-dose liraglutide, when combined with caloric restriction and increased physical activity, has been shown to reduce both visceral fat and liver fat content, as measured by MRI and proton magnetic resonance spectroscopy (^1^H-MRS), respectively, compared with placebo with lifestyle intervention alone in individuals with overweight or obesity at high ASCVD risk. Importantly, these effects were consistent across diverse subgroups stratified by age, sex, race/ethnicity, BMI, and baseline glycemic status, including prediabetes (82). However, it will be challenging in the future to disentangle the independent effects of high-dose semaglutide and tirzepatide per se from the effects attributable solely to weight loss and reduction in ectopic body fat mass.

Moreover, there is ongoing debate regarding the potential negative effects of skeletal muscle mass loss during weight loss, especially in patients with multimorbidity and frailty (83, 84). So far, the available evidence suggests that the muscle mass lost during weight loss with incretin RAs is comparable to other interventions inducing weight loss, such as diet and bariatric surgery, and that it is an adaptive process, comparable to the reduction in LV mass upon arterial blood pressure lowering (84). Overall, despite potential lean mass loss, all CVOTs using long-acting GLP-1RAs resulted in favorable CV outcomes, also independent of age (59).

Beyond metabolic improvements, GLP-1RAs have been shown to exert direct antiatherogenic effects in preclinical studies as discussed above. In humans, the incretins GLP-1 and GIP as well as the GLP-1RA and GLP-1R/GIPR dual agonists have demonstrated antiinflammatory effects (65, 85). For example, liraglutide has been shown to reduce TNF-α and IL-1 production in peripheral mononuclear cells (86). Several GLP-1RAs reduce circulating C-reactive protein (CRP) levels, supporting the hypothesis that reduced inflammation plays a key role in the ASCVD benefit profile of this drug class (52, 59).

In summary, GLP-1RAs reduce ASCVD risk in individuals with T2D, and semaglutide reduces ASCVD in people with obesity, with consistent reductions in MACE observed across multiple CVOTs. These benefits appear to be driven by a combination of modest improvements in CV risk factors (e.g., weight loss, glycemia, blood pressure, lipids, and body weight) and direct vascular and antiinflammatory effects that stabilize plaques and reduce atherogenesis. Therefore, GLP-1RAs have defined a new paradigm in the treatment of metabolic and CV disease — not only improving glycemic parameters but also serving as vascular protective agents.

Ongoing trials, including SURPASS-CVOT, will further delineate the role of GLP-1RAs and combined GLP-1RAs/GIPRAs in ASCVD secondary prevention, including in populations without diabetes. These findings may ultimately expand the indications for GLP-1RA therapy and solidify their role in precision CV risk reduction, for example in different subphenotypes of prediabetes and T2D.

### Clinical studies with focus on heart failure.

A recent meta-analysis of CVOTs of long-acting GLP-1RAs in individuals with T2D showed — in addition to a 14% reduction in MACE (HR, 0.86; 95% CI, 0.81–0.90) and a 12% reduction in all-cause mortality (HR, 0.88; 95% CI, 0.82–0.93) — that treatment with GLP-1RA reduced hospitalization for heart failure (HHF) by 14% (HR, 0.86; 95% CI, 0.79–0.93) (58). The number of individuals with heart failure at baseline varied in the trials, but data from some of these studies suggest that the benefit on heart failure events might be independent of the baseline presence of heart failure. Given the high risk of individuals with T2D and chronic kidney disease (CKD) to develop heart failure as well as the impaired prognosis of patients with all three comorbidities, data from the FLOW trial are of particular interest (87). A prespecified analysis of the FLOW trial evaluated the composite time to the first occurrence of heart failure events (including new onset or worsening heart failure that resulted in an unscheduled hospital admission or urgent visit) or CV death. Among the 3,532 participants in the FLOW trial, 19.2% (678 individuals) had heart failure at baseline, with 47.9% classified as having HFpEF, 18.1% as having heart failure with reduced ejection fraction (HFrEF), and 33.9% with unknown status. In this analysis, semaglutide was found to reduce the risk of composite heart failure events by 27% (HR, 0.73; 95% CI, 0.62–0.87; *P* = 0.0005). This reduction was consistent and significant for both heart failure events and CV death when compared with placebo; heart failure events alone showed an HR of 0.73 (95% CI, 0.58–0.92; *P* = 0.0068), while CV death alone had an HR of 0.71 (95% CI, 0.56–0.89; *P* = 0.0036). The observed effects were consistent across subgroups and regardless of participants’ history of heart failure. It is noteworthy that overall serious adverse events were higher in participants with a history of heart failure compared with those without such a history. Thus, once-weekly administration of semaglutide at a dose of 1.0 mg reduced the risk of heart failure events and CV death in a high-risk population comprising individuals with T2D and CKD, irrespective of their prior history of heart failure (88).

### GLP-1RA studies in HFrEF.

Three small randomized controlled trials have investigated the effects of GLP-1RAs in patients with HFrEF. In the LIVE trial, 241 participants with chronic stable HFrEF, both with and without T2D, were randomly assigned to receive either placebo or liraglutide. After 24 weeks of treatment, there were no observed changes in LV ejection fraction (LVEF), quality of life, or functional class; however, a higher incidence of serious adverse cardiac events (including sustained ventricular tachycardia, atrial fibrillation requiring intervention, and worsening ischemic heart disease) was reported in the liraglutide group compared with placebo (10% vs. 3%, *P* ≤ 0.05) (89). The FIGHT trial involved 300 patients with HFrEF who had experienced a recent HHF, randomly assigned to receive either liraglutide or placebo. After 180 days, the primary outcome — which included time to death, time to rehospitalization for heart failure, and time-averaged proportional change in NT-proBNP levels from baseline — was not different between groups. Furthermore, there was no significant difference in rehospitalizations for heart failure between the two groups (90).

The third trial involved only 82 patients and evaluated the effects of albiglutide versus placebo over a period of 12 weeks in individuals with HFrEF. This study found no significant differences in LVEF, results from a six-minute walk test, myocardial glucose utilization, or oxygen consumption (91). Overall, interpretation of the clinical outcomes is limited due to the small sample size and short duration of the study. These small trials have raised some concerns about the use of GLP-1RAs, but given the results from the CVOTs discussed above, current guidelines summarize GLP-1RAs as a treatment option in patients with T2D and heart failure with proven safety (49).

### GLP-1RA studies in HFpEF.

The growing prevalence of HFpEF is well recognized. Among the various HFpEF phenotypes identified, the obesity-related phenotype — characterized by visceral adiposity and insulin resistance — appears to be the most prevalent (92). This phenotype is associated with impaired physical function, reduced quality of life, and remains an area of unmet therapeutic need. Therefore, it has been hypothesized that semaglutide, given its beneficial effects on body weight, inflammation, and cardiometabolic risk factors, may represent a promising therapeutic approach for obese individuals with HFpEF. In the STEP-HFpEF trial, 529 individuals with HFpEF and BMI ≥30 kg/m^2^ were randomized to 2.4 mg semaglutide weekly versus placebo for 52 weeks. Semaglutide improved heart failure–related symptoms and physical limitation measured by the Kansas City Cardiomyopathy Questionnaire – Clinical Summary Score (KCCQ-CSS), exercise function measured by 6-minute walking distance, and reduced inflammation and body weight (93). These results mark a meaningful step forward in the management of the obesity-related HFpEF phenotype. It should be emphasized, however, that the STEP-HFpEF trial was not designed or powered to assess clinical events, and the impact of semaglutide on “hard” endpoints in heart failure remains to be determined. Still, these findings identified an important therapeutic role for semaglutide in the evolving treatment landscape of heart failure. A similar trial (STEP-HFpEF DM) has been conducted in individuals with T2D and HFpEF, demonstrating similar beneficial results (94).

More recent evidence on the effect of incretin-based therapies in HFpEF comes from the SUMMIT trial assessing the effect of the dual GLP-1RA/GIPRA tirzepatide versus placebo in 731 patients with heart failure, an ejection fraction of at least 50%, and a BMI of at least 30 kg/m^2^. The primary endpoints included a composite of adjudicated CV death or worsening heart failure events and changes in the KCCQ-CSS; 9.9% of patients in the tirzepatide group experienced adjudicated CV death or worsening heart failure events compared with 15.3% in the placebo group (HR, 0.62; 95% CI, 0.41–0.95; *P* = 0.026). Additionally, improvements in KCCQ-CSS scores were higher in the tirzepatide group. Adverse events leading to discontinuation were more common in the tirzepatide group (6.3%) than in the placebo group (1.4%) (95). These data suggest a lower risk of serious CV events and improved health status among tirzepatide-treated patients with heart failure and obesity compared with placebo.

## Conclusions

Collectively, a plethora of preclinical studies have demonstrated that GLP-1 and its RAs exert direct cardioprotective, vascular protective, and antiinflammatory effects that complement their metabolic actions. These mechanistic findings provide a biological foundation for the CV benefits observed in clinical trials and support further exploration of novel incretin–based therapies across cardiometabolic disorders. Overall, preclinical and clinical data align in demonstrating CV protective effects of GLP-1RAs — particularly with respect to vascular protection and heart failure (predominantly HFpEF), while some uncertainties remain regarding the magnitude of benefit on HFrEF-related endpoints and the specific mechanistic pathways involved.

While preclinical studies have provided important mechanistic insights into the CV effects of GLP-1RAs, many experimental models of atherosclerosis, MI, or heart failure were conducted in mice that, despite being fed an HFD or HCD, do not develop the severe obesity or T2D that characterize patients treated with GLP-1RAs in CVOTs. Although several recent preclinical studies using obese or diabetic models have demonstrated broadly consistent cardioprotective effects in line with CVOTs with GLP-1RAs, wider implementation of obesity/diabetes experimental models will be crucial to strengthen the translational link between preclinical mechanisms and clinical outcomes. Future work should therefore validate key pathways under conditions that better reflect the metabolic context of patients with ASCVD or heart failure.

GLP-1RAs were initially developed as agents to control blood glucose in individuals with diabetes, but CVOTs over the last decade have established these medications as a cornerstone for the reduction of CV events in high-risk populations. Various guidelines now give clear recommendations for the use of GLP-1RAs in the management of CVD in individuals with T2D, CKD, and obesity, thus extending the “tool box” for health care providers to reduce CV risk in these patient populations. Given the growing number of individuals with these comorbidities, the main task is to ensure implementation of guideline-directed medical therapy in an interdisciplinary approach.

## Funding support

- Deutsche Forschungsgemeinschaft (DFG; German Research Foundation) TRR 219; Project IDs 322900939 (M03, M05) (to NM), GRK2816 and BI1292/12-1 (to ALB), 520275106 (to the Emmy Noether Research Group), and SFB TRR 219 - 322900939 - M-07 (to FK).
- German Ministry of Education and Science (BMBF) via the German Center for Diabetes Research (DZD e.V.) (to ALB).
- European Research Area Network on Cardiovascular Diseases (ERA-CVD and BMBF, grant no. JTC-2019, MyPenPath - 01KL2004) (to FK).
- European Foundation for the Study of Diabetes (EFSD)/Novo Nordisk Foundation grant NNF2OSA0066111 (to FK).
- Interdisciplinary Centre for Clinical Research within the faculty of Medicine at the RWTH Aachen University (IZKF project PTD 1-11) (to FK).

## Figures and Tables

**Table 1:**
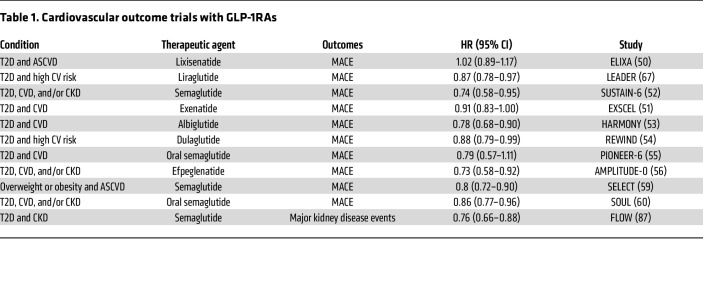
Cardiovascular outcome trials with GLP-1RAs

## Version 1 02/16/2026

Electronic publication
